# Investigation of Vitamin D Status, Age, and Body Mass Index as Determinants of Knee Osteoarthritis Severity Using the Kellgren-Lawrence Grading System in a Saudi Arabian Cohort: A Cross-Sectional Study

**DOI:** 10.7759/cureus.47523

**Published:** 2023-10-23

**Authors:** Meaad Elbashir, Nasser Shubayr, Azhar Alghathami, Sara Ali, Ali Alyami, Neda Alumairi, Ali Abdelrazig, Awatif M Omer, Ohood Elbasheer

**Affiliations:** 1 Department of Diagnostic Radiography Technology, College of Applied Medical Sciences, Jazan University, Jazan, SAU; 2 Department of Radiology, King Abdul Aziz Specialist Hospital, Taif, SAU; 3 Department of Diagnostic Radiology Technology, College of Applied Medical Sciences, Taibah University, Almadinah, SAU; 4 Department of Radiology, Olaya Polyclinic Complex, Riyadh, SAU

**Keywords:** kellgren-lawrence grading system, klgs, x-ray radiography, overweight, vitamin d, knee osteoarthritis

## Abstract

Background

Knee osteoarthritis (OA) is a common and disabling condition affecting millions worldwide. This cross-sectional study endeavors to investigate the relationship between vitamin D status, age, body mass index (BMI), and knee OA in a cohort of individuals in Saudi Arabia.

Methods

In this cross-sectional study, we assessed vitamin D serum levels, conducted knee radiographs, and evaluated the severity of knee OA using the Kellgren-Lawrence grading system (KLGS). The analysis incorporated both descriptive and inferential statistics, including chi-square tests and a regression model to investigate the relationship between KLGS grades as indicators of knee OA severity and vitamin D levels, considering demographics as covariants.

Results

The study included 93 participants with suspected knee OA, of which a substantial portion of the sample population presented with knee OA (58 [62.4%]). Knee OA exhibited a higher prevalence among females, comprising 47 (50.54%) of the total, while 11 (11.83%) were male. The largest age group with knee OA was those older than 58 years, 27 (29.03%), followed by the age group of 48-58 years, 19 (20.43%). Obesity was a prevalent factor among knee OA patients (36 [38.7%]), with grade 2 (17 [18.3%]) and grade 3 (24 [25.8%]) being the most frequent. Vitamin D deficiency was prevalent in 54 (58%) of patients. Among knee OA cases, bilateral involvement was predominant in 46 (79%), with a substantial portion, 36 (62%), presenting deficient vitamin D levels. The regression model revealed that age (95% CI: 0.54-1.03, p < 0.001) and BMI (95% CI: 0.01-0.60, p = 0.04) significantly predict higher KLGS grades, indicating that increasing age and higher BMI are associated with higher KLGS grades. However, Vitamin D levels did not show a significant impact on the severity of knee OA.

Conclusions

The findings from this study highlight the importance of monitoring and maintaining adequate vitamin D levels to potentially reduce the risk of knee OA and the need for early detection and intervention to manage knee OA, particularly in females, older poplulation, and obese adults. They may guide healthcare providers in developing comprehensive approaches to reduce the risk of this condition.

## Introduction

Knee osteoarthritis (OA) stands as a prevalent and incapacitating musculoskeletal ailment, emerging as one of the primary causes of pain, disability, and diminished quality of life worldwide [1]. Characterized by the gradual deterioration of articular cartilage in the knee joint, knee OA affects millions of individuals, contributing significantly to the global burden of chronic diseases [2]. As the aging population continues to expand and obesity rates continue to rise, the incidence and impact of knee OA are expected to escalate, demanding a deeper understanding of its risk factors and etiological underpinnings [3-5].

Numerous risk factors have been implicated in the development and progression of knee OA, with vitamin D status, age, and body mass index (BMI) emerging as pivotal contributors to this complex interplay. Recent research endeavors have spotlighted an intricate relationship between these factors and knee OA, highlighting the need for comprehensive investigations to elucidate their roles and interactions [2,6-8].

Vitamin D, a fat-soluble vitamin synthesized in the skin upon exposure to sunlight and obtained from dietary sources, has garnered considerable attention in the context of musculoskeletal health [3,9,10]. Beyond its well-established role in calcium homeostasis and bone metabolism, emerging evidence suggests that vitamin D has immunomodulatory, anti-inflammatory, and chondroprotective effects, all of which could potentially influence the development and progression of knee OA [11,12]. Deficiency in this essential nutrient has been linked to various musculoskeletal disorders, prompting investigations into its association with knee OA [13].

Age represents an indisputable risk factor for knee OA. As individuals age, the likelihood of developing knee OA increases substantially, with degenerative changes in the articular cartilage, subchondral bone, and synovial membrane becoming more pronounced [6,13,14]. The intricate mechanisms underlying age-related OA remain an area of active research, encompassing cellular senescence, oxidative stress, chronic low-grade inflammation, and alterations in extracellular matrix components [13,14].

BMI, reflecting the relationship between weight and height, is another critical factor implicated in knee OA. Excessive body weight places substantial mechanical stress on weight-bearing joints such as the knees, potentially accelerating cartilage degradation and OA progression [15,16]. Epidemiological studies have consistently reported a relationship between obesity and knee OA, with higher BMI levels corresponding to an elevated risk of disease development and greater OA severity [8,17]. In addition to being a risk factor for OA, obesity is also associated with a higher risk of vitamin D deficiency. This is because vitamin D is stored in fat cells, and obese individuals may need more vitamin D to maintain adequate levels [10]. X-ray radiography is a commonly used tool for diagnosing knee OA and can also assess body weight and bone density [14]. By correlating the results of X-ray radiography with vitamin D levels, we can gain insights into the relationship between obesity, vitamin D, and knee OA [14].

Despite the mounting evidence regarding these risk factors, a comprehensive understanding of their interactions and relative contributions to knee OA in Saudi Arabia remains elusive. Furthermore, the influence of these factors in a population with increasing rates of obesity has not been widely explored. To address these knowledge gaps, this cross-sectional study endeavors to investigate the relationship between vitamin D status, age, BMI, and knee OA in a cohort of individuals in Saudi Arabia. Consequently, this study holds particular significance as it contributes valuable insights into the etiology and potential preventive strategies for knee OA, offering a unique perspective within the context of the Saudi Arabian population. As the burden of knee OA continues to mount globally, this research has the potential to inform clinical practice, guide public health policies, and, ultimately, improve the quality of life for individuals afflicted by this debilitating condition.

## Materials and methods

Study design and population

This prospective cross-sectional study was conducted from August 2022 to April 2023 in Taif City, Saudi Arabia. The study involves assessing the vitamin D status of participants using blood samples, measuring their BMI, and performing knee X-ray radiography to assess the severity of knee OA. The sample size was calculated based on an effect size of 0.5, a p-value of 0.05, and a power of 0.8, using G*Power software. The minimum calculated sample size was at least 94 participants. A total of 93 participants with suspected knee OA were recruited by convenience sampling method. Participants were eligible if they were adults aged 18 years and above, had no history of knee surgeries or fractures, and were willing to undergo blood tests and X-ray radiography. We excluded children, pregnant women, and patients with a history of previous knee surgeries.

Serum vitamin D measurement

A laboratory test called a 25-hydroxy vitamin D (25(OH)D) test was conducted to measure serum vitamin D from a blood sample. This test measures the amount of 25-hydroxy vitamin D in the blood, the body's main circulating form of vitamin D. The test was performed using a serum sample using automated immunoassay techniques. Serum vitamin D measurements were classified into "deficient" and "normal" categories, with a cutoff point of less than 20 ng/mL (50 nmol/L) indicating deficiency [18].

Knee radiograph assessments

The radiography technique used to assess knee OA involved obtaining weight-bearing anteroposterior (AP) and lateral knee radiographs. These radiographs were taken with the patient standing upright and the X-ray beam directed at a fixed angle to the knee joint. During the procedure, the patient was positioned with the knee joints positioned against the X-ray cassette or detector. The X-ray machine was then adjusted to obtain the clearest images possible, and the radiographs were taken. The radiographic images obtained are then evaluated by a radiologist using KLGS.

To determine the extent of OA, the radiographs can be examined using the KLGS, which evaluates the severity of the condition based on factors such as the narrowing of the joint space, the existence of osteophytes, and other radiographic features. The KLGS ranges from 0 to 4, with each grade indicating different levels of osteoarthritic changes in the joint:

Grade 0: No evidence of OA.

Grade 1: Doubtful narrowing of the joint space and possible presence of osteophytes (bone spurs).

Grade 2: Definite osteophytes and possible joint space narrowing.

Grade 3: Multiple osteophytes, definite joint space narrowing, and possible sclerosis (hardening) of bone.

Grade 4: Large osteophytes, significant joint space narrowing, and other extreme degenerative changes indicating severe OA.

The radiologist interpreted X-ray images of the affected joint and assigned the appropriate KLGS grade based on the observed radiographic features. This grading system helps in assessing the severity of OA, guiding treatment decisions, and monitoring disease progression.

Ethical considerations

Ethical clearance for the study was gained from the Institutional Review Board of the Ethical Committee in King Abdul Aziz Specialist Hospital (KAASH), Taif, Saudi Arabia (IRB No: HAP-02-T-067/228). Informed consent was obtained from the study participants prior to study commencement.

Statistical analysis

Statistical analysis was conducted using the Statistical Package for the Social Sciences (SPSS) Version 26 (IBM Corp., Armonk, NY). Descriptive statistics were used to summarize categorical data, presenting frequencies and percentages. To assess associations between variables, chi-squared tests were performed. Additionally, a logistic regression analysis was conducted to examine the relationship between KLGS grades and vitamin D levels, with age and BMI as covariates. The significance level for all inferential tests was set at p < 0.05.

## Results

The total number was 93 patients, of whom 18 (19.4%) were males and 75 (80.6%) were females, with an age range of 25 to 70 years. The frequency distribution for patient age groups was as follows: 10 (10.8%) were aged 25-36 years, 25 (26.9%) were aged 37-47 years, 27 (29%) were aged 48-58 years, and 31 (33.3%) were older than 58 years. As for BMI, there were four groups: underweight, normal, overweight, and obese. The percentages of patients in each group were as follows: three (3.2%) were underweight, 13 (14%) were normal weight, 24 (25.8%) were overweight, and 53 (57%) were obese. Knee OA exhibited a higher prevalence among females, comprising 47 (50.54%) of the total, while 11 (11.83%) were males. The largest age group with knee OA was those aged over 58 years (27 [29.03%]), followed by the age group of 48-58 years (19 [20.43%]). Regarding BMI among those with knee OA, the majority fell into the obese category, accounting for 36 (38.70%) of the sample. Overall, a substantial portion of the sample population presented with knee OA (58 [62.4%]) compared to those without the condition (35 [37.6%]). The association of demographic variables with knee OA revealed significant associations between age groups and BMI categories with knee OA (Table 1).

**Table 1:** Frequency distribution for patients’ gender, age and BMI for all patients and association with knee OA BMI, body mass index; OA, osteoarthritis

Variables		N (%)	Knee OA		P-value
			No	Yes	
Gender	Male	18 (19.4)	7 (7.53)	11 (11.83)	0.193
	Female	75 (80.6)	28 (30.11)	47 (50.54)	
Age groups (years)	25-36	10 (10.8)	8 (8.6)	2 (2.15)	<0.001
	37-47	25 (26.9)	15 (16.13)	10 (10.75)	
	48-58	27 (29)	8 (8.6)	19 (20.43)	
	>58	31 (33.3)	4 (4.3)	27 (29.03)	
BMI	Underweight	3 (3.2)	2 (2.15)	1 (1.08)	0.036
	Normal	13 (14)	8 (8.6)	5 (5.38)	
	Overweight	24 (25.8)	8 (8.6)	16 (17.2)	
	Obese	53 (57)	17 (18.3)	36 (38.70)	
Total			35 (37.6)	58 (62.4)	

Table 2 shows the frequency distribution for vitamin D levels and KLGS for all patients. Regarding vitamin D levels, 54 (58%) patients had deficient levels, while 39 (42%) had normal levels. KLGS was used to assess the severity of knee OA, with 35 (37.6%) of patients having grade 0 and 58 (62.4%) having grades 1-4, with grade 3 representing the largest portion of the sample 24 (25.8%).

**Table 2:** Frequency distribution for knee OA and vitamin D level using the Kellgren-Lawrence grading system.

Variables		N	Percent (%)
Vitamin D level ng/mL	Normal	39	42.0
	Deficient	54	58.0
Kellgren-Lawrence grading system	Grade 0	35	37.6
	Grade 1	8	8.6
	Grade 2	17	18.3
	Grade 3	24	25.8
	Grade 4	9	9.7

**Figure 1:**
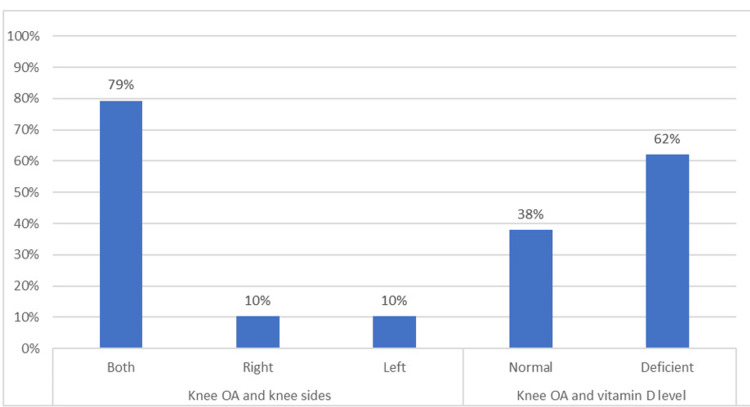
Crosstabulation between the vitamin D level with knee OA and knee joint sides. Figure 1 shows that most participants had knee OA in both knees. Regarding vitamin D levels, the majority of those with knee OA had deficient vitamin D levels.

Figure 1 illustrates the distributions of knee OA between vitamin D levels and joint side among the study participants. In terms of knee sides, the majority of individuals with knee OA experienced arthritis in both knees, accounting for 46 (79%) of cases. Meanwhile, six (10%) cases were associated with the right knee and another six (10%) with the left knee. Regarding vitamin D levels, among those with knee OA, a substantial 36 (62%) had deficient vitamin D levels, while 22 (38%) had normal vitamin D levels.

In this logistic regression model examining factors influencing KLGS grades as indicators of OA severity, age (95% CI: 0.54-1.03, p < 0.001) and BMI (95% CI: 0.01-0.60, p = 0.04) were found to be significant predictors with positive coefficients, indicating that increasing age and higher BMI are associated with higher KLGS grades. However, the contrast between normal and deficient vitamin D levels did not show statistical significance (95% CI: -0.86 to 0.13, p = 0.15) in relation to KLGS grades (Table 3).

**Table 3:** Analysis of the regression model between KLGS grades and vitamin D level, with age and BMI as covariates. t, t-test statistic; SE, standard of error KLGS, Kellgren-Lawrence grading system

					95% Confidence Interval	
Predictor	Estimate	SE	t	p-Value	Lower	Upper
Intercept	-0.41	0.44	-0.94	0.348	-1.28	0.46
Age	0.79	0.12	6.34		0.54	1.03
BMI	0.31	0.15	2.08	0.04	0.01	0.6
Vitamin D level						
Normal – Deficient	-0.36	0.25	-1.45	0.15	-0.86	0.13

Table 3 demonstrates significant positive coefficients between knee OA severity and age (95% CI: 0.54-1.03, p < 0.001) and BMI (95% CI: 0.01-0.60, p = 0.04). However, deficient vitamin D levels did not show statistical significance (95% CI: -0.86 to 0.13, p = 0.15).

## Discussion

The current study provides valuable insights into the prevalence and factors associated with knee OA within the studied population. Our findings reveal a substantial burden of knee OA, with 58 (62.4%) of patients affected, and age and BMI demonstrated a significant association with knee OA, while gender did not exhibit a significant association with knee OA in our study. Also, our study shows that 53 (57%) of knee OA patients were obese, 24 (25.8%) were overweight, and 16 (17.2%) were of normal or underweight.

Kim et al. found that the prevalence of knee OA in a community-based elderly population in Thailand was 35.4%, with a higher prevalence in females and those with a higher BMI [19]. Another study from India reported an overall prevalence of knee OA of 28.7%, with factors such as female gender, obesity, age, and sedentary work being associated with a higher prevalence [8]. Cui et al. conducted a global meta-analysis and reported a pooled global prevalence of knee OA of 16.0% in individuals aged 15 years and above and 22.9% in individuals aged 40 years and above [2]. A study by Grotle et al. reported that the prevalence of knee OA was significantly higher in obese individuals than in those with normal weight and that the risk of knee OA increased with increasing BMI [1]. Similarly, a study found that obese individuals were three times more likely to develop knee OA than those with normal weight [3]. Excess weight places additional stress on weight-bearing joints such as the knees, potentially accelerating the degenerative process.

The result of this study showed that 46 (79%) of knee OA patients had it in both knees, and 12 (10%) had it in the right knee and the left knee each. Previous studies have suggested that unilateral knee OA is more common in younger adults, while bilateral knee OA is more common in older adults [9]. Conversely, another study indicated that bilateral knee OA was more common in adults aged 50 years and older, while unilateral knee OA was more common in younger adults [20].

Regarding vitamin D levels, most (54 [58%]) patients had deficient levels, while 39 (42%) had normal levels. Several previous studies indicated a high prevalence of vitamin D deficiency in different populations. Forrest and Stuhldreher analyzed data from the U.S. population and reported an overall prevalence rate of 41.6% for vitamin D deficiency, with higher rates among blacks (82.1%) and Hispanics (69.2%) [21]. In Saudi Arabia, a systematic review reported a prevalence of 81.0% of vitamin D deficiency among different populations [22], while another study reported 29% of their study sample being deficient [23]. The findings from Figure 1 indicate that a substantial majority, specifically 36 (62%) of patients, had knee OA when their vitamin D levels were insufficient. This observation underlines a potential connection between vitamin D deficiency and the occurrence of knee OA in the study population. A previous study observed lower vitamin D levels in knee OA patients compared to controls, and a significant progression of medial meniscal grading in OA patients with vitamin D levels <10 ng/mL [24]. Another study found that vitamin D deficiency was associated with an increased risk of knee OA and that vitamin D supplementation may have beneficial effects on knee OA [16].

The regression analysis further explored the relationship between KLGS grades and vitamin D levels, with age and BMI as covariates. Age and BMI emerged as significant predictors of KLGS grades, with higher age and BMI associated with higher KLGS grades, indicative of greater OA severity. Previous studies have yielded inconsistent results concerning the role of age, BMI, and vitamin D levels as predictors that influence the severity of knee osteoarthritis, as measured by the KLGS. Avasthi et al. identified age, and BMI as predictors of knee OA severity [25]. Several previous studies found no significant association between vitamin D levels and the severity of radiographic knee OA [7,11]. On the other hand, other studies have suggested an association between vitamin D deficiency and knee OA progression [6,26].

Limitations

There are several limitations to consider in the interpretation of the results. First, the study had a relatively small sample size, which may have limited the statistical power to detect significant differences in certain variables. Second, the study was conducted in a specific patient population and may not be generalizable to other populations. Third, the study was cross-sectional in nature, which limits the ability to establish causality or temporal relationships. Fourth, the study did not account for other factors that may influence the development and progression of knee OA, such as physical activity or genetics. These limitations highlight the need for further research with larger sample sizes, more diverse patient populations, longitudinal study designs, and more comprehensive measures of potential risk factors for knee OA.

## Conclusions

Our findings provide valuable insights into the factors associated with the occurrence and severity of knee OA in this specific cohort. The study revealed that knee OA is more prevalent among females and those aged over 58 years. Additionally, obesity emerged as a substantial risk factor for knee OA, with a majority of knee OA patients falling into the obese category. The overall prevalence of knee OA in our sample was noteworthy, affecting about two-thirds of participants. Vitamin D deficiency was a common finding in our study, impacting a significant proportion of the patients. Intriguingly, while the study highlighted the significance of age and BMI as predictors of more severe knee OA, it did not establish a significant correlation between vitamin D levels and OA severity. These findings emphasize the importance of monitoring and maintaining adequate vitamin D levels, particularly among older adults, females, and those at risk of obesity. Early detection and intervention strategies are crucial in managing knee OA effectively. Healthcare providers should consider comprehensive approaches that address the specific risk factors present in their patient populations.
